# An Internet of Things-Oriented Adaptive Mutation PSO-BPNN Algorithm to Assist the Construction of Entrepreneurship Evaluation Models for College Students

**DOI:** 10.1155/2021/3371383

**Published:** 2021-12-15

**Authors:** Huaxiang Fu

**Affiliations:** Zhejiang Industry Polytechnic College, Shaoxing, Zhejiang 312000, China

## Abstract

In this paper, the IoT-based adaptive mutation PSO-BPNN algorithm is used to conduct in-depth research and analysis of the entrepreneurship evaluation model for college students and practical applications. This paper details the principle, implementation, and characteristics of each BP algorithm and PSO algorithm. When classifying college students' entrepreneurship evaluation based on BP neural network, because BP algorithm is a local optimization-seeking algorithm, it is easy to fall into local minima in the training phase of the network and the convergence speed is slow, which leads to the reduction of classifier recognition rate. To address the above problems, this paper proposes the algorithm of PSO optimized BP neural network (PSO-BPNN) and establishes a classification and recognition model based on this algorithm for college students' entrepreneurship evaluation. The predicted values obtained from the particle swarm optimization neural network model are used to calculate the gray intervals, and the modeling samples are further screened using the gray intervals and the correlation principle, while the hyperspectral particle swarm optimization neural network model of soil organic matter based on the gray intervals is established afterward; and the estimation results are compared and analyzed with those of traditional modeling methods. The results showed that the coefficient of determination of the gray interval-based particle swarm optimization neural network model was 0.8826, and the average relative error was 3.572%, while the coefficient of determination of the particle swarm optimization neural network model was 0.853, and the average relative error was 4.34%; the average relative errors of the BP neural network model, support vector machine model, and multiple linear regression model were 8.79%, 6.717%, and 9.9%, respectively. The average relative errors of the BP neural network model, support vector machine model, and multiple linear regression model are 8.79%, 6.717%, and 9.468%, respectively. In general, the entrepreneurial ability of college students is at a good level (83.42 points), among which the entrepreneurial management ability score (84.30 points) and entrepreneurial spirit (84.16 points) are basically the same, while the entrepreneurial technology ability is relatively low (82.76 points), and the evaluation results are further verified by the double case analysis method. The current problems encountered by university students in entrepreneurship are mainly the lack of practicality, which indicates that universities, industries, and national strategy implementation levels are not sufficiently focused and collaborative in entrepreneurship development to varying degrees.

## 1. Introduction

With the birth and growth of the Internet, all kinds of related technologies develop at a high speed, the distance between people is drawn closer and closer, and work and life are increasingly inseparable from the Internet. Internet of Things (IoT) technology, as an important branch of Internet technology, combines various information sensing devices with the Internet, completes the transmission and sharing of data through the Internet, and uses computer technology to realize the processing and application of relevant data [1]. This technology extends the information exchange once limited to human to human, to things to people, and to things to things, making the future network more intelligent and realizing the interconnection of all things. With the development of the information technology revolution and the extension of economic globalization, “innovation” has increasingly become a core element in promoting social and economic development. To meet the trend of the times, the Party and the government have made important strategic plans for building an innovative country, and “innovation” will be the main theme for a long period. Innovation needs to be led by direction, and for this reason, the development paths of “independent innovation”, “integrated innovation”, and “re-innovation after introduction, digestion, and absorption” have been put forward one after another. To achieve innovation, it is necessary to cultivate and shape many innovators and entrepreneurs who meet the needs of the times [2]. Clarified the relationship between the five major elements of academic evaluation and the subjective and objective factors of creativity training, as well as the mechanism of their influence, through theoretical analysis and empirical research, pointed out that strong chemical industry evaluation's guidance for creativity training is for creativity training, and it is of great significance. How to improve the innovation and entrepreneurial competency of college students has become the focus of research in the academic field. The purpose of this paper is to construct a theoretical model of entrepreneurial competency that is consistent with the characteristics and rules of entrepreneurship development of college students by drawing on existing research results and empirical studies and to empirically evaluate and test the overall level and influencing factors of entrepreneurial competency of college students through the theoretical model, to contribute to the in-depth development of college students' entrepreneurship research and practice [3].

The scientific evaluation of the entrepreneurial competencies of science and technology students and the effective guidance of their entrepreneurial activities are theoretical issues that need to be addressed urgently. Entrepreneurship as a serious socio-economic activity has its logic and laws, and entrepreneurial behavior is in line with the consensus of economics and management, that is, entrepreneurship as a market-oriented behavior requires that entrepreneurs should have a competence structure that meets the needs of entrepreneurship, and it is absolutely difficult to guarantee the smooth development and profitability of entrepreneurs by ignoring the competence elements and to ensure that the country's strategic goal of achieving social stability and economic development through high-quality entrepreneurship of college students can be successfully achieved [4]. It is difficult to ensure that the strategic goal of social stability and economic development can be successfully achieved through the high-quality entrepreneurship of college students. Therefore, the systematic answer to the questions of what is the basic competency structure of entrepreneurship among college students, whether contemporary college students have entrepreneurial competency, the degree of proficiency in their entrepreneurial competency, and how to improve college students' entrepreneurial competency has become a major issue that cannot be avoided in the field of entrepreneurship research. How to effectively identify and construct a competency structure system is not only an inherent requirement of entrepreneurship evaluation for science and technology undergraduates but also an objective need to effectively guide their entrepreneurial activities.

The structural contradiction between university graduates and market demand is increasingly prominent. The talents cultivated by the old training model cannot match the requirements of the economic transformation on the quality and knowledge skills of talents, resulting in the embarrassing situation that enterprises cannot obtain effective talent replenishment in the labor market and college graduates can hardly meet the requirements of job positions. Thus, the participation of college students in entrepreneurship is an important means to enhance their comprehensive quality and improve their social survival ability, and it is also an effective way to drive employment through entrepreneurship and relieve social employment pressure. Entrepreneurial practice allows college students to have a deeper contact with society and understand it, to discover problems, stimulate innovation, solve problems, and create value. For college students, school is only a small classroom for learning cultural knowledge, but society is the big classroom that can bring more learning and growth space for college students. Entrepreneurial practice is like a bridge between school and society, which can greatly accelerate the process of professionalization and socialization of college students and improve their comprehensive ability to adapt to society. To sum up, the era has given innovative, practical, humanistic, and social characteristics to university students' entrepreneurship. It is these characteristics of the times that reflect the importance of guiding and enhancing students' entrepreneurial competence and stimulating and tapping their potential human capital in the context of “building an innovative country, promoting the deepening reform of higher education, and transforming the development connotation of the market economy”.

## 2. Current Status of Research

A model of entrepreneurial process based on the view of entrepreneurship as organizational creation is proposed, which considers that the entrepreneurial process can be divided into two stages: first, the preorganizational venture formation period, in which entrepreneurial activity is mainly expressed as individual entrepreneurial behavior; second, the postorganizational venture operation period, in which entrepreneurial activity is mainly expressed as organizational behavior of the entrepreneurial enterprise [5]. Some scholars, on the other hand, divide the entrepreneurial process into five stages according to the growth process of a new enterprise: first, the proof-of-principle stage, where the main task is to verify the feasibility of the innovative technology; second, the prototype stage, where the product is produced and the prototype organizational structure is formed; third, the model sales stage, where the main task is to improve the feasibility of the product and make the necessary professional division of labor for financial, marketing, and other operational activities; fourth, the start-up stage, where the profitability of the product further increases, the organizational structure expands further, and more product and management issues arise; and fifth is the natural growth stage, where the organization enters natural expansion and the entrepreneur usually considers new strategic adjustments or enters a new entrepreneurial cycle [6]. Avoid harming the person being evaluated. It is necessary to explore how to cultivate students' creativity in academic evaluation. This is a subject that spans two research fields. Naturally, it is inseparable from the research of creativity cultivation. The existence stage, the main goal is to make business opportunities to form enterprises, where only a small number of enterprises can earn enough cash income to enter the second stage; survival stage, where the goal is to grow the business into the next stage and cannot stagnate in the survival stage for long enough to cause cash flow to dry up; is the success stage, where the organization is taking shape and the business is earning good revenues and needs to adjust its strategy in time to prevent the business from regressing in the face of external changes; the takeover stage, where investors look for opportunities to take the business off the table and voluntary or involuntary changes in management occur; and the resource maturity stage, where the business is operating with abundant resources and effective realization of economies of scale [7].

It is proposed that the entrepreneurial process is a highly dynamic balance between the appropriate allocation of entrepreneurial opportunities, entrepreneurial team, and resources, and entrepreneurial opportunities, resources, and entrepreneurial team are the key components of the entrepreneurial process [8]. In the early stage of entrepreneurship, the key is the exploration and selection of opportunities, and the decision of the entrepreneurial team is focused on the rapid integration of resources to seize the entrepreneurial opportunities; as the new enterprise is founded and grown, resources become more abundant, and the enterprise faces a more complex competitive environment and market environment, the decision of the entrepreneurial team is focused on the rational allocation of resources to improve the efficiency of resource use, and the construction of a standardized management system to resist external competition and uncertainty [9]. A theoretical model of the entrepreneurial process based on the interaction between the entrepreneur and the new enterprise is proposed, arguing that the entrepreneur and the new enterprise are the key components of the entrepreneurial process and that the entrepreneurial process is essentially a process of close interaction between the entrepreneur and the new enterprise under the action of the external environment [10]. The only way to make entrepreneurship research more systematic based on diversification is to gradually build a framework for entrepreneurship research independent of other fields, and then provide a more comprehensive longitudinal research on entrepreneurial competency, entrepreneurial ability, and another individual level [11].

The entrepreneurial process concretely expressed as the act and activity of achieving stage equilibrium in a constant dynamic change. In the entrepreneurial process, there is never absolute stability and equilibrium, and any enterprise and organization are in dynamic change to seek temporary unity and coordination of the elements of entrepreneurship. In different stages of development, entrepreneurs need to analyze the problems and risks that may arise in the process of development from the perspective of dynamic balance and make changes and deployment of their capabilities and organizational resources according to the time and the actual situation, to achieve sustainable development of the enterprise.

## 3. Adaptive Mutation of the Internet of Things PSO-BPNN Algorithm-Assisted Evaluation of College Student Entrepreneurship Analysis

### 3.1. IoT Adaptive Mutation PSO-BPNN Algorithm Design

Random access (RA) refers to the process in which the UE or terminal sends an access request to the access control system, and the system allocates access resources such as access channels and time-frequency resource blocks after a certain algorithm and feeds this information back to the terminal, while the data transmission process generally arranged after obtaining the allocated access resources [12]. Random-access methods can be divided into two categories based on the different resource allocation methods: competition-based random-access methods and noncompetition-based random-access methods. The biggest difference between competition-based and noncompetition-based random-access methods is the different allocation of the preamble codes, competition-based random-access method by the UE side to initiate the application of the preamble codes, and the UEs do not know whether the application of the preamble codes is duplicated, so there is a problem of collision, while noncompetition-based random-access method by the access control side of the unified allocation of the preamble codes, so there is no possibility of conflict. The access process also does not require the steps of applying for the leading code and competition resolution.

**Figure 1:**
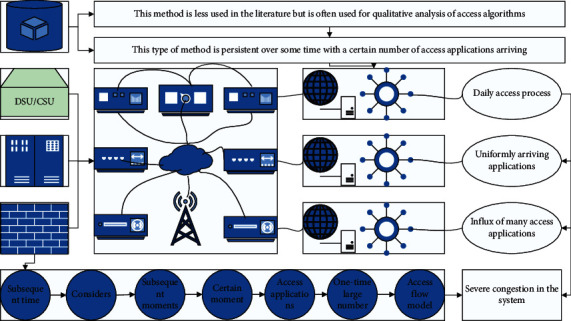
IoT adaptation mutation framework.

In the daily access process, uniformly arriving applications often do not cause congestion, and only the influx of many access applications in a short period will lead to severe congestion in the system. Therefore, to simplify the user access flow model, some literature only considers the impact of a one-time large number of arriving access applications on the system and only considers the one-time arrival of N MTC devices at a certain moment in the simulation, without considering whether the subsequent moments. The simulation only considers the arrival of N MTC devices at one time and does not consider whether there are further arrivals of new access requests at subsequent times [13]. This method is less used in the literature but is often used for qualitative analysis of access algorithms, such as the impact of different leading code values on the system access performance. This type of method is persistent over some time with a certain number of access applications arriving, and the specific number of arrivals per time slot can conform to different distribution rates. This type of access flow model is more in line with the actual access in use and is more dynamic and continuous than the first type of access flow model. Depending on the arrival rate of each time slot, this type of access flow model can also be divided into several categories, as shown in Figure 1. At the same time, it also pays attention to the improvement of the comprehensive quality and practical ability of innovation and entrepreneurship, especially the expansion of self-entrepreneurship awareness and innovative operation ability, so that students can find and solve problems independently, and then put forward their own new views and opinions, for the life of students The continuous development of learning and the individual has laid a solid foundation.

In the IoT mass access scenario, for periodic services such as the regular reporting of sensor data is suitable for uniformly distributed or Poisson distributed access flow model, while for bursty services such as alarm-like services is suitable for beta distributed access flow model, IoT as an emerging application in the communication industry, driven by big data and artificial intelligence, the market scale will further expand, with technological progress [14]. With technological advancement, government support, and increasingly improved industry standards, China's IoT industry will continue its good development momentum and provide new impetus for sustained and stable economic growth. The wave of expansion of mobile Internet to the Internet of everything will create larger market space and industrial opportunities compared to the Internet [9]. IoT has become an important technical support for many high-tech enterprises in China, such as cloud computing, mobile payment system, bicycle-sharing, and other IoT-related industrial chains, and have achieved great success in the Chinese market, bringing good economic benefits while also bringing great convenience to our travel and work.

Network sizing estimation is usually a comprehensive analysis of both coverage and capacity to determine the size, i.e., the number of sites, required for network planning in each area. On the one hand, the coverage estimation combines the link budget with the propagation model to calculate the coverage radius of the base stations to find the number of base stations required to cover the planning area; on the other hand, the capacity estimation calculates the number of base stations required to meet all the service demands by processing various actual services into some virtual equivalent services based on the resources provided by individual base stations. The larger of the coverage estimate and the capacity estimate are used as the number of sites for the size estimate.

(1) $$\begin{array}{c} R_{\text{SBS}}=\left\{d_{sll}|\text{PL}\left(d_{su}\right)=\chi_{\text{PL}}^{2}\right\},\\ N_{\text{cap}}=\sum_{k=1}^{N_{\text{region}}}N_{\text{cap}}^{k}\chi_{\text{PL}}^{2}. \end{array}$$

Among the various types of artificial neural networks, multilayer feedforward networks can not only solve the heterogeneous problem and the arbitrary function approximation problem but also the classifier constructed based on this network has a strong classification recognition ability. BPNN is a typical feedforward neural network, but it has slow convergence and weak generalization ability. Many practical problems have many local minima due to the complex multidimensional nature of the problem itself, and because the BP algorithm itself uses the gradient descent method to find the optimal value will also lead to local minima, resulting in the overall design of the network is more likely to fall into local minima. To solve the above problems of convergence and local minima in BPNN, this paper proposes to optimize the training neural network with the help of a particle swarm algorithm.

The PSO algorithm optimizes the BPNN by replacing the gradient descent method to correct the network weights and thresholds. The key point to consider in this process is to construct a mapping between the neural network weights and thresholds and the particle dimensions of the PSO algorithm. The key to the training of the BPNN is the continuous correction of the weights and thresholds, while the PSO algorithm is the iteration of the individual particle positions and velocities [15]. Therefore, when the two algorithms are combined, the weights and thresholds of the BP algorithm correspond to the particle positions of the PSO algorithm, and the total number of weights and thresholds is the dimensionality of the particle population. The fitness function of the particles is generally chosen as the objective function, so the fitness function of the particles is determined as MSE according to the end condition of the BP algorithm as the reference minimum mean square error (MSE).

(2) $$\begin{array}{c} \text{MSE}=\frac{1}{P}\sum_{p=1}^{p}\overset{m}{\underset{k=1}{\sum }}{\left(T_{k}^{p}+Y_{k+1}^{p}\right)}^{2}. \end{array}$$

In this paper, the principle of the PSO-BPNN algorithm is to optimize the parameters of BPNN by particle swarm algorithm training, mainly optimizing the weights and thresholds, which can effectively solve the problem of BPNN falling into local miniaturization and slow convergence, and improve the training efficiency of the network. PSO algorithm in training optimization of BPNN, the weights, and thresholds of BPNN is derived from the position vector of particles. In the search process of the particles, the minimum is reached using the following equation:

(3) $$\begin{array}{c} {\text{Fitness}}_{i}=\frac{1}{N}\sum_{i=1}^{p}\overset{m}{\underset{j=1}{\sum }}{\left(W_{ij}^{d}-W_{ji}^{p}\right)}^{2}. \end{array}$$

**Figure 2:**
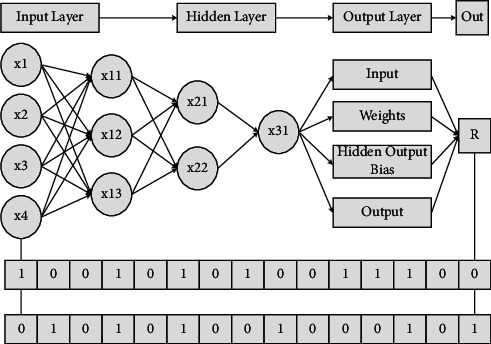
PSO-BPNN algorithm framework.

Innovation and entrepreneurship education is oriented to all students, emphasizing not only the cultivation of basic literacy, innovation spirit, entrepreneurial consciousness, and entrepreneurial ability but also the improvement of comprehensive quality and practical ability of innovation and entrepreneurship, especially the expansion of self-entrepreneurial consciousness and innovative operation ability, so that students can independently identify problems and solve them, and then put forward their new views and opinions, which lay a solid foundation for students' lifelong learning and individual. The weights and thresholds of the BP algorithm correspond to the particle positions of the PSO algorithm, and the total number of weights and thresholds is the dimension of the particle swarm. This provides students with a solid foundation for lifelong learning and sustainable development [16]. The purpose of innovation and entrepreneurship education is not to let every student start a business, but the core is to cultivate students' innovation spirit, entrepreneurial consciousness, and entrepreneurial ability, focus on the cultivation of students' independent learning and practical ability, closely combine the cultivation of talents with scientific research and the needs of society, and change from the traditional classroom focus on knowledge transfer to the emphasis on ability and comprehensive quality cultivation, which can effectively improve the quality of talent cultivation in colleges and universities, as shown in Figure 2.

Educational evaluation should be as impartial and objective as possible to make the results of the evaluation have a greater reference value. In the development of educational evaluation activities, objective facts should be used as the basis, and the reference standard for evaluation should be used as the yardstick, eliminating as far as possible the interference of the evaluator's subjective factors and external objective factors in the evaluation. Both the methods and means of evaluation and the standards of evaluation should be in line with the norms of ethics and morality. Once the evaluation criteria have been established, they should be strictly enforced from the beginning to the end. In addition, access to evaluation information should be justified and the use of evaluation results should be legal to avoid harming those being evaluated. To explore how to cultivate students' creativity in academic assessment, which is a topic that spans two research fields, it is natural that the research on creativity cultivation is inseparable. There are many research results, both theoretical and practical, on creativity education and creativity cultivation. Analysis of the components of creativity and its characteristics can provide a more scientific theoretical basis for carrying out academic assessment reform.

### 3.2. Design of Entrepreneurship Evaluation Model for College Students in Higher Education

**Figure 3:**
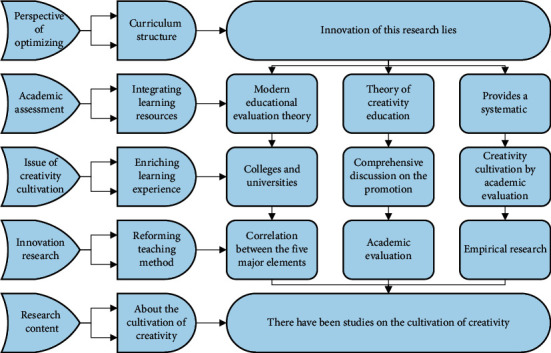
Evaluation hierarchy model.

The innovation of this research lies in: first, the innovation of research perspective. There have been studies on the cultivation of creativity, mainly from the perspective of optimizing curriculum structure, integrating learning resources, enriching learning experience, reforming teaching methods and approaches to talk about the cultivation of creativity, but this study starts from academic assessment to discuss the issue of creativity cultivation, which is an innovation in terms of research perspective. Second, it is an innovation in research content. Using the modern educational evaluation theory and the theory of creativity education, this study provides a systematic and comprehensive discussion on the promotion of creativity cultivation by academic evaluation in colleges and universities, clarifies the correlation between the five major elements of academic evaluation and the subjective and objective factors of creativity cultivation, as well as the mechanism of their influence [17]. Through theoretical analysis and empirical research, it is pointed out that strengthening the orientation of academic evaluation on creativity cultivation is of great significance to creativity cultivation, summarizing the academic evaluation reform strategy of “five guides,” constructing a framework system for academic evaluation of college students, and proposing specific operational suggestions for the reform of evaluation contents and evaluation methods of different types of coursework. In the analysis of the problem by AHP, the relevant influencing factors and solutions are listed according to the overall objective of the problem, and a hierarchical model is constructed. The constructed hierarchical model generally consists of three levels, namely, the objective level, the criterion level, and the solution level. The criterion layer, also known as the intermediate layer, represents the factors that need to be considered and the guidelines for decision making to achieve the overall objective and is the intermediate link to achieve the overall objective, as shown in Figure 3.

The elements of the evaluation of graduate students' innovation and entrepreneurship education initially formulated in this study are mainly analyzed in terms of top-level design, teachers, students, and educational environment and forms. After consulting experts and synthesizing their opinions, the top-level design mainly designed two evaluation dimensions, namely, the incentive support policies related to graduate students' innovation and entrepreneurship and the evaluation of universities' attention to graduate students' innovation and entrepreneurship education. The study explores the influencing factors of postgraduate innovation and entrepreneurship and then designs a framework of postgraduate innovation and entrepreneurship education evaluation system with high realistic guidance value. Moreover, the classifier based on this network has strong classification and recognition capabilities. Therefore, exploring the optimization problem of feedforward neural networks has practical significance for the research in many fields.

**Figure 4:**
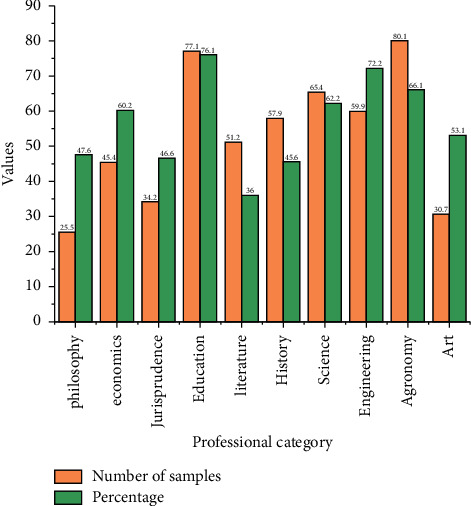
Structural distribution of the questionnaire sample.

The support of graduate student innovation and entrepreneurship education and the teaching methods of graduate student innovation and entrepreneurship education, coded as DA1, DA2, and DA3 in order; the measurable indicators of faculty provision are the faculty provision of graduate student innovation education and the faculty provision of graduate student entrepreneurship education, coded as IF1 and IF2 in order; and the measurable indicators of implementation methods are the practical forms of graduate student innovation education and the practical forms of graduate student entrepreneurship education. Based on the questionnaire survey, to further explore the relationship between the influencing factors, a theoretical model on graduate innovation and entrepreneurship education was constructed with the help of structural equation modeling, as shown in Figure 4.

After running the standardized estimates calculation, the results of the unstandardized path coefficients of the model show that all the path coefficients except the 11 path coefficient reference indicators reach a significant level of 0.05, the measurement errors are positive, the absolute values of the critical ratios are greater than 1.96, and the significance probability values are less than 0.001, indicating that all the path coefficients are significant and the constructed [18]. The structural equation theoretical model constructed is not problematic. The overall index of graduate students' information literacy is 52.4, which is consistent with the current situation of graduate students' innovation and entrepreneurship education, indicating that the indexes of the influencing factors of graduate students' innovation and entrepreneurship education selected in this study are more effective. However, the value of the total index is only slightly greater than 50, which is not a high index, reflecting that the current situation of the development of innovation and entrepreneurship education for graduate students is not optimistic. The existing evaluation structure of entrepreneurial competency of college students is mainly derived from the evaluation model of entrepreneurial competency of Western entrepreneurs, which is thinly localized on the one hand; and on the other hand, it is difficult to apply the entrepreneurial competency model of general entrepreneurs to college students' entrepreneurs.

**Figure 5:**
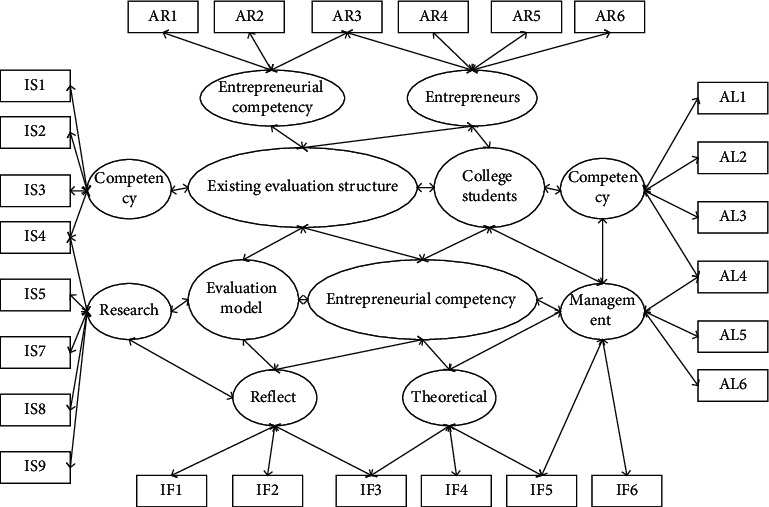
Theoretical path diagram of the structural equation model.

Moreover, the current entrepreneurial competency research not highly differentiated from the competency research, which makes it difficult to reflect the theoretical depth of entrepreneurial competency research. Such a research pattern is not conducive to the scientific development of entrepreneurship among college students in science and technology, nor is it conducive to the scientific decision making of management agencies [19]. To determine the scale required for network planning in a given area, that is, the number of sites. Coverage estimation should combine the link budget with the propagation model, calculate the coverage radius of the base station, and find the number of base stations required to cover the planned area. This study precisely seeks to make a breakthrough in this field, deepen theoretically the understanding of entrepreneurial competence, dig deeper into the influencing factors of entrepreneurial competence and countermeasures of cultivation from the theory of college students' entrepreneurship, and strengthen the theoretical explanatory power of evaluation research with vivid cases and an appropriate amount of quantitative research methods to form a theoretical research structure combining depth and breadth, as shown in Figure 5.

This study takes the evaluation of the ability of science and technology students' entrepreneurship as the research theme, studies the current operation mechanism of entrepreneurship education, constructs a system of ability characteristics based on the comprehensive use of scientific methods, relies on field research and quantitative analysis to process, and organize the system, finally constructs an evaluation index system and mathematical model, and verifies it through empirical methods. The main research methods used include literature research method, questionnaire survey method, statistical analysis method, case study method, expert interview method, etc. Each method is not applied independently, but fully cooperated, repeatedly used, and mutually corroborated. Through this study, we can deepen the theoretical depth of college students' entrepreneurial ability evaluation research, expand the field of college students' entrepreneurial ability evaluation research to the group of science and technology college students, and propose a model of evaluation of college students' entrepreneurial ability in science and technology with the characteristics of the times, reflecting the spirit of China and in line with the reality of science and technology college students and verify it [20]. The biggest potential problem at this stage is that some team members may have palpitations about the difficulties of the turbulent period and cannot express their opinions and insights effectively and sincerely. Guiding and encouraging members to let go of their psychological burdens, delegating a certain degree and scope of authority, and focusing on nurturing team members' responsibility, sense of mission, and trust are the main focuses of team leaders. In terms of mechanism design for implementing the path, a reasonable and effective system of incentives, constraints, and penalties should be gradually established and improved.

## 4. Analysis of Results

### 4.1. IoT Adaptation Mutation PSO-BPNN Algorithm Results

**Figure 6:**
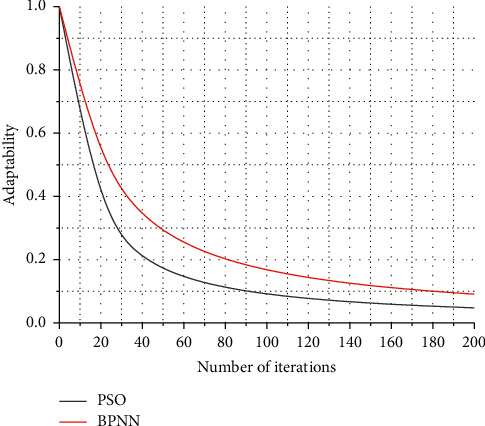
Iterative adaptation degree value graph.

In the initialization of the particle swarm, the particle swarm size *M* = 60, the particle swarm size is too small will not be obvious for the optimization of the later BPNN, when *M* is less than 50, PSO optimization of BPNN will still exist easy to fall into the condition of local minima, when *M* is greater than 50 then PSO optimization of BPNN is obvious. The scale of the market will be further expanded. With technological progress, government support, and increasingly perfect industry standards, the Internet of Things industry will continue to maintain a good momentum of development and provide new impetus for sustained and stable economic growth. The sum of the number of weights and thresholds in BPNN is 305, so this particle population has dimension *D* = 305, and usually the fitness function is the training error function MSE of the BPNN. The number of iterations is defined as 200, the learning factors *c*, and care both 2, and the inertia weight is 0.5. The output of the PSO algorithm is stored as weights and thresholds to facilitate the training of the BPNN in the next step. The PSO algorithm is selected instead of the gradient descent method to correct the weights and thresholds and start training the BPNN so that the training accuracy reaches 0.00001 or the defined number of training times 1000 times ends with a training learning rate of 0.1. When the net training is learned, the feature vectors of any 30 groups of samples for each Chinese character are mixed for training, the total number of training sample images is 960, and the final training samples are built for the next test recognition. The PSO algorithm optimizes the iterative fitness values for BPNN, as shown in Figure 6.

**Figure 7:**
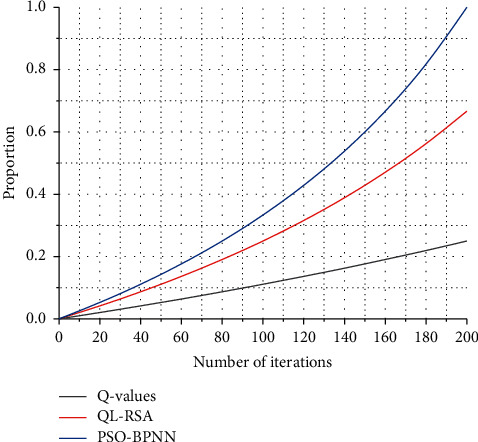
Percentage of ideal throughput accounted for by different algorithms.

Figure 6 shows that the performance is only improved by about 3% compared to randomly selecting a single relay for transmission, which is much lower than the QL-RSA algorithm under DF protocol. It can be seen from Figure 7 that the performance of the QL-RSA algorithm is not much optimized with random selection as the total transmit power increases. Specifically, the arrival amount of each time slot can conform to different distribution rates. This type of access flow model is more in line with the actual access situation and is more dynamic and continuous than the first type of access flow model. This is because the amplified forwarding protocol amplifies the transmit power of the useful signal at the second hop in addition to the amplified noise power, thus resulting in the received signal-to-noise ratio at the destination of each node is not very different, and the received signal-to-noise ratio is used as a reward for Q-learning, then the difference in the Q-value of each node is not too big, and the Softmax selection strategy is precisely based on the corresponding *Q*-value of each node. The Softmax selection strategy is based on the corresponding *Q*-value of each node to make a decision. This is the reason the performance of the QL-RSA algorithm is not much optimized than that of R-RSA.

This is because when the signal-to-noise ratio required at the receiving end is not high, anyone relay node can meet the requirements at the receiving end, so the number of nodes for the three algorithms does not differ much. When the total number of relays is larger, the number of relays required for cooperative transmission is smaller, because the more densely distributed the relays are, the better the combination of the selected set of relays can be, so that a larger signal-to-noise ratio can be achieved at the receiving end, which in turn means that fewer relays can be used to achieve the upper bound on the signal-to-noise ratio at the receiving end. The optimal single-relay selection and the multirelay selection are discussed separately for the two forwarding protocols. The data transmission process is generally arranged after the allocated access resources are obtained. Random-access methods can be divided into contention-based random-access methods and noncompetition-based random-access methods according to different resource allocation methods. As a comparison of simulation results, this paper also simulates the results of the R-RSA algorithm and analyzes the performance of the QL-RSA algorithm from different perspectives of average throughput, the probability distribution of selected nodes, and accounting for the ideal average throughput, the simulation results show that the performance of QL-RSA is obvious compared to R-RSA when DF forwarding protocol is used, but the effect is not so obvious for AF forwarding protocol.

### 4.2. Results of the Evaluation of Entrepreneurship among College Students in Higher Education

**Figure 8:**
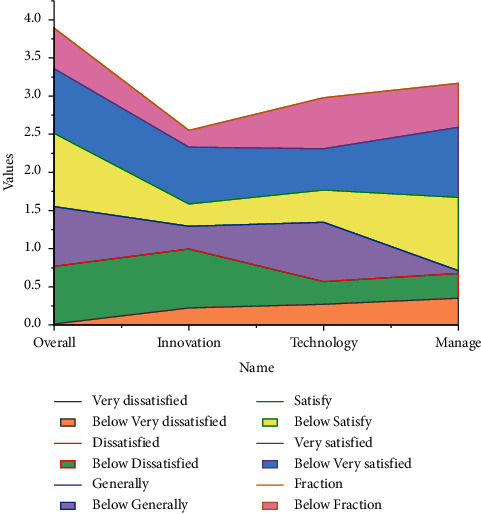
College students' entrepreneurship scores.

In general, the entrepreneurial ability of science and technology college students is at a good level, among which entrepreneurial management ability and entrepreneurship are equal, and entrepreneurial technology ability is relatively low. The entrepreneurial technology ability of science and technology college students is the center of gravity in their entrepreneurial ability structure, but it is also a relative shortboard that still has room for improvement. The above phenomenon can be tentatively explained by referring to Timmons' entrepreneurial process theory. While solving its own employment, it creates more opportunities and jobs for the society. The participation of college students in entrepreneurship is an important means to enhance their own comprehensive quality and improve their social viability, and it is also an effective way to promote employment through entrepreneurship and relieve social employment pressure. In the early stage of entrepreneurship, entrepreneurs are required to have deep and good professional abilities. For science and technology undergraduates, the level of technical ability can largely influence their quality of exploring and grasping opportunities, while the improvement of technical ability is quite difficult. The influence of scientific and technological ability on entrepreneurial management ability and entrepreneurial spirits, such as resource application and team management, is weak, and such management ability and entrepreneurial spirit are less required in the early stage of entrepreneurship, and college students can basically reach the initial response after a few years of systematic higher education, so the evaluation is good. However, it should be noted that in the midstage of entrepreneurship, the requirements for resource application and management skills are substantially increased, and the reorganization of resources, the re-creation of opportunities, and the operation of teams are raised to a strategic level. The subjects observed in this study are mainly university students, which are in line with the competence performance of subjects in the early or even pre-entrepreneurial stage of entrepreneurship, as shown in Figure 8.

Teaching induction requires teachers to accept and adopt new teaching methods. In entrepreneurship teaching activities for science and technology college students, teachers can use task-driven and typical demonstration teaching methods to induce students to spontaneously generate entrepreneurial views and consolidate entrepreneurial enthusiasm to develop entrepreneurial ability. In the task-driven segment, teachers set specific tasks with technical points and difficulties of technology-based entrepreneurial activities, ask students to complete the tasks in groups and give comments and scores according to the process and results of the groups' completion of the tasks, which induces students' enthusiasm for competition and teamwork, and gains professional knowledge and experience in the process of completing the tasks.

**Figure 9:**
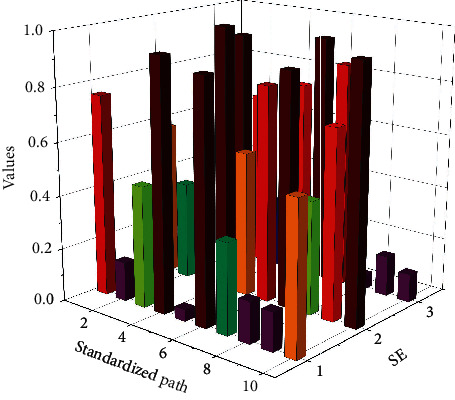
Path coefficients of the modified entrepreneurial opportunity capability structure model.

In the typical demonstration, session teachers introduce skilled entrepreneurs or R&D leaders in enterprises into the school to teach the more practical and operational aspects of the teaching. These entrepreneurs are successful models of real entrepreneurship, and interactive exchanges with entrepreneurs can induce students' entrepreneurial enthusiasm perceptually, effectively stimulate students' sense of imitation, consolidate students' enduring interest in entrepreneurship, and enhance the bonding of entrepreneurship education. To enhance the effectiveness of teaching induction, we can consider organizational restructuring by establishing a special entrepreneurship center or entrepreneurship college to cooperate with the technological innovation centers, product development centers, and employment counseling agencies that are currently prevalent in universities, to bring into play the entrepreneurship education and counseling functions of these organizations. It has very important theoretical value and innovative significance for enriching and perfecting the evaluation theory of graduate students' innovation and entrepreneurship education. For example, the Entrepreneurship Center is responsible for providing resources for research and development of entrepreneurship courses, strengthening links with engineering and technology-related industries and academia through cooperation with science and technology transformation departments, and publishing horizontal research topics on innovation and entrepreneurship. It can also cooperate with on-campus employment counseling agencies to provide students with entrepreneurial counseling and practical platform services in terms of industry and career development prospects to enhance students' confidence in entrepreneurship, as shown in Figure 9.

To further illustrate the relationship between the factors of entrepreneurship development resources and entrepreneurial competencies, a structural equation model was conducted separately in this chapter to test the relationship between entrepreneurship development resources and entrepreneurial generic competencies, entrepreneurial professional competencies, and entrepreneurial opportunity competencies, and the results showed that the effects of the factors on entrepreneurship generic competencies and entrepreneurial professional competencies were social network resources, practical training resources, education and teaching resources, and policy and cultural resources, respectively. How to improve the systematic response to practical problems such as college students' entrepreneurial ability has become a major issue that cannot be avoided in the field of entrepreneurship research. The results showed that the effects of the factors on entrepreneurial general ability and entrepreneurial professional ability were social network resources, practical training resources, education and teaching resources, and policy and cultural resources, respectively, and the effects of the factors on entrepreneurial opportunity ability were social network resources, education and teaching resources, and practical training resources, respectively, and policy and cultural resources did not have an effective effect on entrepreneurial opportunity ability.

## 5. Conclusion

This paper outlines the principle, structure, and implementation process of BPNN and its properties. The PSO-BPNN algorithm is proposed to optimize the BPNN with the PSO algorithm for the defects of slow convergence and easy to fall into the local minima of BPNN. And the prediction feasibility of the optimized algorithm is verified by function simulation fitting, and the optimized PSO-BPNN algorithm has the advantages of high prediction accuracy, fast convergence, and fewer training times than the original BPNN algorithm. Based on the predicted value of the application volume and based on the principle of the maximum number of successful next time slot accesses, the leading code grouping threshold and the ACB control parameters are adjusted, and then the newly arrived service types are identified according to the QoS requirements and the size of the data volume, and the previously derived parameters are used, the access method of time slot Aloha is adopted for the services with insensitive delay and small effective data volume and the cultivation of entrepreneurial ability of college students. The purpose is to build a set of competency theoretical models that conform to the characteristics and laws of college students' entrepreneurial development by referring to existing research results and empirical research. This study shows that the effect of the factors on entrepreneurial general ability and entrepreneurial professional ability is social network resources, practical training resources, education and teaching resources, and policy and cultural resources, respectively; the effect of the factors on entrepreneurial opportunity ability is social network resources, education and teaching resources, and practical training resources, respectively.

## Data Availability

The data used to support the findings of this study are available from the corresponding author upon request.
